# Theoretical designing of non-fullerene derived organic heterocyclic compounds with enhanced nonlinear optical amplitude: a DFT based prediction

**DOI:** 10.1038/s41598-022-21894-x

**Published:** 2022-11-23

**Authors:** Muhammad Khalid, Mashal Khan, Khalid Mahmood, Muhammad Arshad, Muhammad Imran, Ataualpa Albert Carmo Braga, Riaz Hussain

**Affiliations:** 1 Institute of Chemistry, Khwaja Fareed University of Engineering & Information Technology, Rahim Yar Khan, 64200 Pakistan; 2 Centre for Theoretical and Computational Research, Khwaja Fareed University of Engineering & Information Technology, Rahim Yar Khan, 64200 Pakistan; 3 Institute of Chemical Sciences, Bahauddin Zakariya University, Multan, 60800 Pakistan; 4 Department of Chemical Engineering, College of Engineering, King Khalid University, Abha, Saudi Arabia; 5 Department of Chemistry, Faculty of Science, King Khalid University, P.O. Box 9004, Abha, 61413 Saudi Arabia; 6 Departamento de Química Fundamental, Instituto de Química, Universidade de São Paulo, Av. Prof. Lineu Prestes, 748, São Paulo, 05508-000 Brazil; 7 Department of Chemistry, Division of Science and Technology, University of Education , Lahore, Pakistan

**Keywords:** Chemistry, Optics and photonics

## Abstract

In current era, non-fullerene (NF) chromophores have been reported as significant NLO materials due to promising optoelectronic properties. Therefore, a series of NF based chromophores abbreviated as **TPBD2-TPBD6** with D–π–A architecture was designed from the reference compound (**TPBR1**) by its structural tailoring with an efficient donor and various acceptor groups for the first time. First, the structures of said compounds were optimized at M06-2X/6-311G (d,p) level. Further, the optimized structures were utilized to execute frontier molecular orbitals (FMOs), UV–Visible (UV–Vis) absorption, density of states (DOS) and transition density matrix (TDM) analyses at the same level to understand the non-linear (NLO) response of **TPBR1** and **TPBD2-TPBD6**. Promising NLO results were achieved for all derivatives i.e., the highest amplitude of linear polarizability ⟨*α*⟩, first (*β*_total_) and second ($\gamma$_total_) hyperpolarizabilities than their parent molecule. The compound **TPBD3** was noted with the most significant NLO properties as compared to the standard molecule. The structural modeling approach by utilizing the acceptor molecules has played a prominent role in attaining favorable NLO responses in the molecules. Thus, our study has tempted the experimentalists to synthesize the proposed NLO materials for the modern optoelectronic high-tech applications.

## Introduction

Organic compounds possessing second and third-order NLO properties are the subject of great interest because of their various applications in the field of telecommunication and optical data processing^1^. Organic NLO materials show the following characteristics: (i) NLO susceptibilities, (ii) optical clarity, (iii) thermal stability and (iv) solubility via structural modifications. For this purpose, a sufficient knowledge of the molecular structure is required to elucidate the relationship among various parts of the studied compound^2^. The sophisticated modifications in the molecular structures owing to the synthetic tools of organic chemistry result in the fine tuning of optical properties. Intramolecular charge transference (ICT), HOMO–LUMO band gaps, transmission of electron density via π-linkers, electronic dipole moments and excited electronic transition states are the most notable features of NLO materials^3^. HOMO–LUMO band gaps are influenced by extended conjugation of the donor–π–acceptor based compounds which is one of the major characteristics of an organic molecule. For this, suitable donor, π-spacer or acceptor are incorporated to achieve the desired NLO active molecular configuration^4^. The donor–π–acceptor configured organic compounds are reported with remarkable first (*β*_total_) and second ($\gamma$_total_) hyperpolarizabilities^5^.

Among a large variety of organic materials, fullerenes and their derivatives are also regarded as the most effective NLO molecules in addition to their role as an organic solar cell^6,7^. Fullerenes are electron deficient 3-dimensional cage like π-conjugated structures possess active NLO responses due to delocalization of the electronic charge^8^.The fullerene C_60_ derivatives (2a, 2b and 2c) are designed using pyrrolidine and tetrathiafulvalene moieties. Robust second-order and third-order NLO properties are exhibited by compound 2c with *β*_tot_ = 15.69 × 10^–30^ esu and < γ >  = 284.29 × 10^–36^ esu, respectively. The < γ > value was about three times higher than the parent compound. The study revealed the importance of π-conjugation in tuning the NLO properties of donor–π–acceptor fullerene compounds^9^. Though there are several important applications of fullerenes in optoelectronics and in nonlinear optics, however, there are some drawbacks that persist in this early class of compounds. These include; weaker absorption in the visible and NIR regions; thermal and photochemical instability; non-tunable energies of LUMO and less absorption of sunlight. An idea to replace fullerene-based electron acceptors has revolutionized the field of material sciences to a great extent. There is a need of more efficient optoelectronic materials for which non-fullerene acceptors (NFAs) is an emerging area especially in the field of organic semiconductors^10^. They have facile synthesis, wide optical assimilation, suitable structural morphology, tunable band gaps and strong light absorption capabilities as compared to fullerene derivatives^11–15^. Owing to their stable nature, their optoelectronic properties can be modified for obtaining promising results. Khalid et al. designed dipyrrolo [2,3-b:20,30-e]pyrazine2,6 (1H,5H)-dione (PzDP) based small molecular non-fullerene acceptor moieties with A1–π–A2–π–A1 configuration and performed their quantum chemical study. Interestingly, higher open circuit voltage with wider absorption values are recorded. Hence, small molecular NFAs proved as efficient materials for optics and electronics^16^. According to our best knowledge, very few NLO based work has been reported regarding NFAs up till now^10,17^. Not long since, NFAs have appeared an interesting area of modern NLO study and this research paper would be another contribution in elucidating non-linear optical properties of the selected NFA molecule and its derivatives^18^. The literature includes a variety of structural frameworks such as donor–acceptor, donor–π–linker-acceptor, donor–π–acceptor–π–donor, donor–π–π–acceptor, and donor–acceptor–π–spacer–acceptor^19,20^. These push–pull schemes accelerate the range of penetration towards greater wavelengths, decrease the HOMO–LUMO energy differences and extend delocalization of electrons, consequently exhibiting a good NLO behavior. Anna et al.determined the NLO properties of push–pull tetrazoles using hyper-Rayleigh scattering technique with the help of femtosecond Ti:Sapphire laser. The derivative (1d) with the strongest push–pull mechanism is found with highest NLO activity. Moreover, the same compound exhibited the least HOMO–LUMO energy gap (4.97 eV) which leaded to efficient charge transfer^21^. Similarly, another series of push–pull porphyrins having triphenylamine (TPA) and dicyanovinyl (DCN) groups have been synthesized and characterized using various spectroscopic techniques and DFT studies for calculating their third-order NLO properties. Surprisingly, the push–pull chromophores exhibited bathochromic shifts (21–48 nm and 38–80 nm) which is consistent with enhanced resonance due to TPA and *–I* effect of DCN group. The two-photon absorption coefficients (*β*) are found in the range of 0.87 × 10^−13^ to 4.28 × 10^−13^ m W^−1^ while, the nonlinear refractive index (*n*_2_) in the range of 1.21 × 10^−19^ to 7.36 × 10^−19^ m^2^ W^−1^. The results revealed them as potential candidates in the nonlinear optics and photonic devices^22^. Keeping in view the above discussion, herein, we have taken **BDTN-Th** as a parent non-fullerene acceptor molecule^23^ and modified it into a new reference compound (**TPBR1**): 2-((*Z*)-5-((6-((2-((*Z*)-(4-(dicyanomethylene)-6-oxo-4*H*-cyclopenta[c]thiophen-5(6*H*)-ylidene)methyl)-4-isobutyl-4*H*-thieno[3,2-b]pyrrol-6-yl)thiophen)-4-isobutyl-5,8-dimethoxy-4*H*-benzo[4,5]thieno[3,2-b]thieno[2,3-d]pyrrol-2-yl)methylene)-6-oxo-5,6-dihydro-4*H*-cyclopenta[c]thiophen-4-ylidene)malononitrile^23^. After literature review, structural tailoring of **TPBR1** is done by modifying the one end capped acceptor with a strong donor (Dibenzo-Tetraazafulvalene i.e. Dibenzo-TAF)^24^ having an IUPAC name as 14,15-dimethyl-7,8,14,15-tetrahydro-6H-benzo[4,5]imidazo[1,2-a]benzo[4,5]imidazo[2,1-c][1,4]diazepine, and various kinds of strong electron withdrawing acceptor moieties on the other end in order to achieve strong push–pull configuration. A DFT based study for **TPBR1** and its derivatives has been presented in this research paper. It is anticipated that our NLO-based study on NFAs may not only fulfills the above mentioned research gap but also provides a new pathway for researchers in further exploration of NLO behavior.

## Computational procedure

In present investigation, non-fullerene based acceptor type chromophores (**TPBD2-TPBD6**) were designed with D–π–A configuration by structural tailoring with various vigorous acceptor units. The geometries of the **TPBR1** and **TPBD2-TPBD6** were optimized at M06-2X functional^25^ and 6-311G(d,p) basis set at ground state using DFT approach. The 6-311G(d,p) basis set (a split-valence triple-zeta basis plus d, p polarization functions on non-hydrogen and hydrogen, respectively)^26^ was a hybrid method. To exploit the NLO behavior of afore-mentioned chromophores various quantum chemical investigations like frontier molecular orbital (FMO), density of states (DOS) and absorption properties were executed utilizing the Gaussian 09 program package^27^. For the investigation of solvent effect, conductor like polarizable continuum model (**CPCM**) was utilized^28^. A variety of software were employed to get the reliable information of entitled compounds which included Gaussum^29^, Avogadro^30^, Chemcraft^31^, Multiwfn^32^ and Gauss View 5.0^33^ were utilized for interpreting the results from output files. To determine the chemical reactivity of **TPBR1** and **TPBD2-TPBD6**, global reactivity parameters (GRPs) were calculated from their HOMO–LUMO energy band gaps^34^. The quantitative analysis of D–π–A architecture was performed in DOS in order to support the the information obtained by frontier molecular orbitals. The NLO properties like average polarizability ⟨*α*⟩, first hyperpolarizability (*β*_total_)^35^ and second hyperpolarizability ($\gamma$_total_) were estimated as the above-mentioned functional.

## Results and discussion

### Structural designing of D–π–A species

**Figure 1:**
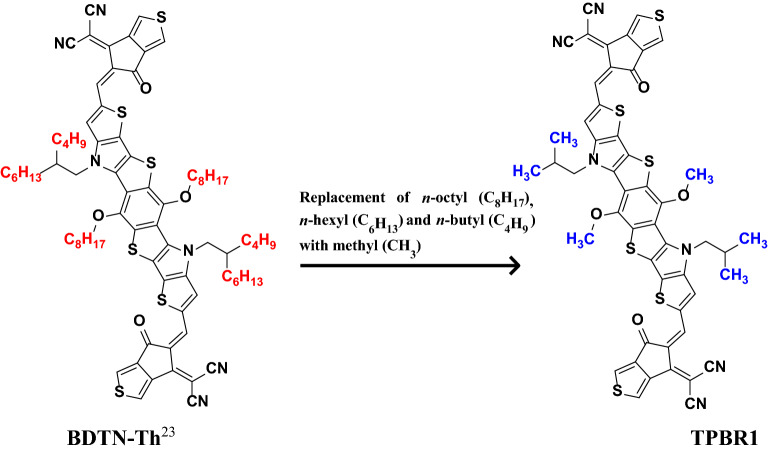
Side-chain modification of parent molecule (**BDTN-Th**)^23^ into a reference (**TPBR1**) molecule. These structures are drawn with the help of ChemDraw software (https://chemistrydocs.com/chemdraw-pro-8-0/).

**Figure 2:**
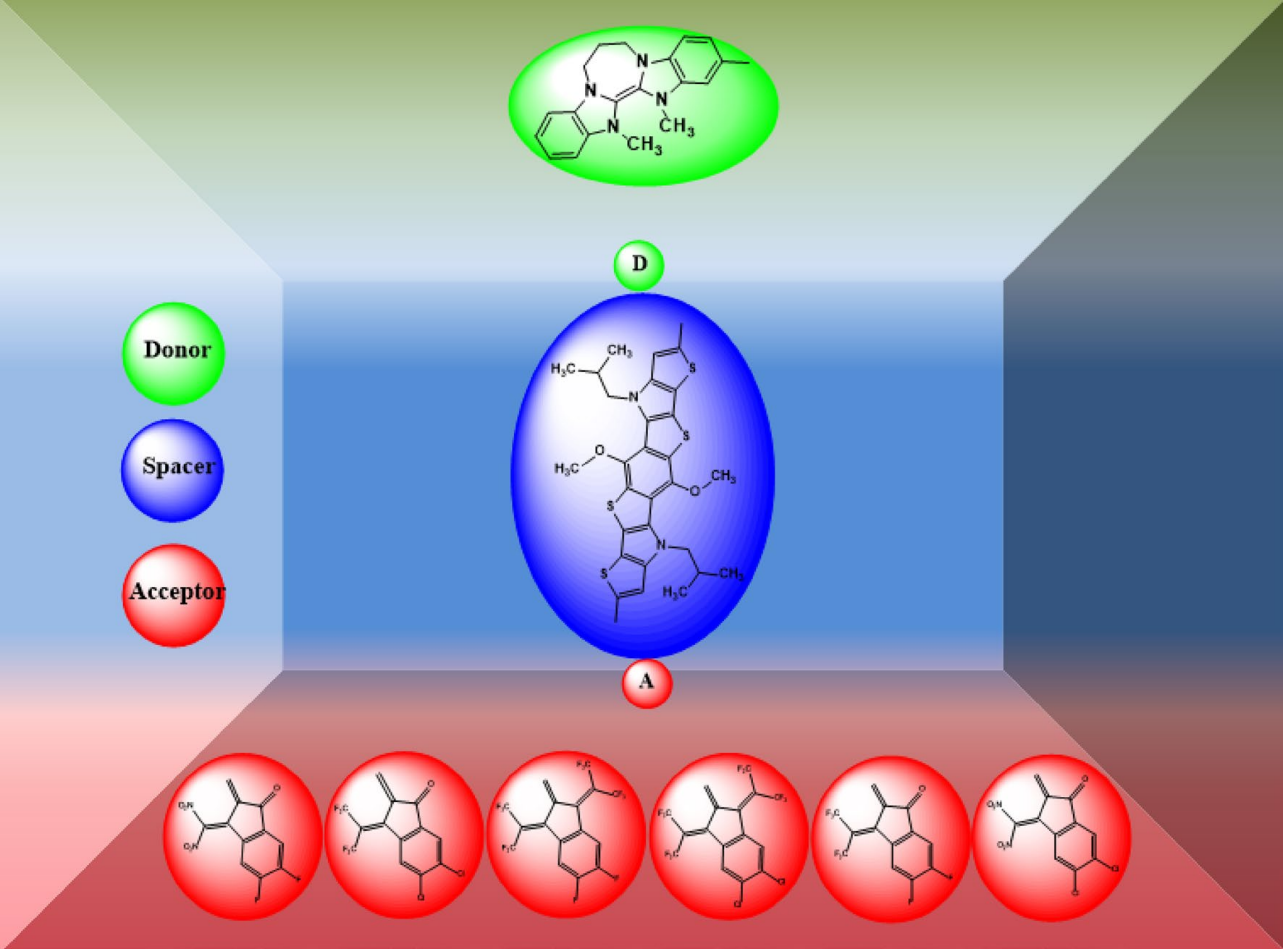
A structural map is consisting of acceptor (red color), spacer (blue color) and donor (green color) of the entitled compounds. These structures are drawn with the help of ChemDraw software (https://chemistrydocs.com/chemdraw-pro-8-0/).

The present quantum chemical study envisages some efficient non-fullerene based D–π–A infrastructures as NLO probes with fused heterocyclic component as an essential part of every structure, are schematically represented in Fig. S1 and the optimized structures are represented in Fig. S2 in the supplementary information (SI). The reference (**TPBR1**) is prepared by side-chain modifications in the parent (**BDTN-Th**) molecule^23^ as shown in the Fig. 1 in order to avoid computational cost. An exceptionally strong donor moiety i.e. Dibenzo-Tetraazafulvalene (Dibenzo-TAF)^24^ is utilized along with a central heterocyclic fused ring structure (functioning as a π–core) are retained in all derivatives. The Dibenzo-TAF has been regarded as one of the “Super-Electron Donors” and acts as an aromatic stabilized cation as well as possesses electron-donating nitrogen atoms both of which can assist in the electron transfer^36^. The compound **TPBR1** is A–π–A type which is structurally modified into D–π–A derivatives (**TPBD2-TPBD6**). The first derivative (**TPBD2**) is formed by introducing the donor moiety at its one acceptor end, while the other end-capped acceptor is retained. The rest of the compounds (**TPBD3**, **TPBD4**, **TPBD5** and **TPBD6**) are designed with same D–π–A configuration by modifying the vigorous acceptor moieties keeping the same donor group as that in **TPBD2**. The Fig. S3 represents the structures of all acceptors along with their IUPAC names which are employed in the structural modeling. A schematic representation of the entitled molecules is presented in Fig. 2. It is anticipated that they may function as innovative NLO systems in research and technology. Moreover, literature survey has revealed that acceptor species are important in tuning the energy band gap (*E*_LUMO-HOMO_) and absorption wavelength (*λ*_max_) of a compound^37^. Therefore, we have designed five derivatives abbreviated as **TPBD2-TPBD6** from a parent chromophore (**TPBR1**).

The following computed parameters demonstrated the influence of acceptor and donor moieties on the NLO response: (i) energy band gap (*E*_gap_); (ii) absorption wavelength (*λ*_max_); (iii) global reactivity parameters like softness, hardness, electronegativity etc.; (iv) density of states (DOS); (v) hole-electron indices; (vi) linear polarizability ⟨*α*⟩; (vii) first hyperpolarizability (*β*_total_) and (viii) second hyperpolarizability ($\gamma$_total_). It is anticipated that our designed chromophores will act as an effective photonic material that expresses marvelous NLO properties.

### Electronic study

**Figure 3:**
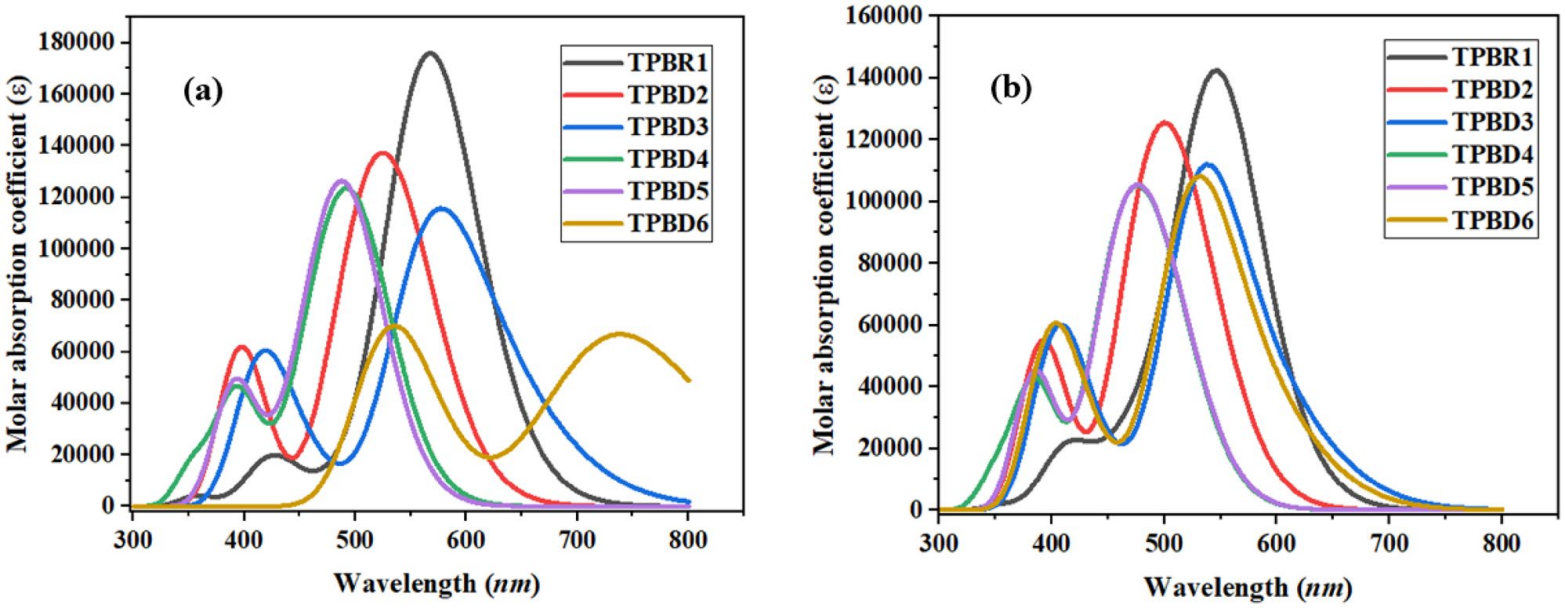
Absorption spectra of entitled compounds in chloroform (**a**) and gas phase (**b**). These graphs are drawn by utilizing Origin Pro 8.5 version (https://originpro.informer.com/8.5/). All out put flies of entitled compounds were computed through Gaussian 09 version D.01 (https://gaussian.com/g09citation/).

The frontier molecular orbitals (FMOs) analysis reveals the electron density distribution pattern for the highest occupied molecular orbital (HOMO) and lowest unoccupied molecular orbital (LUMO)^38^. The Fig. 3 illustrates the electron density over HOMOs and LUMOs of the compounds under discussion. The energy band gap (*E*_LUMO _− *E*_HOMO_) obtained from the said analysis is of prime importance in the quantum chemical investigation of materials possessing non-linear optical properties^39,40^ are shown in Table 1. The main orbitals (HOMO/LUMO) are shown in Fig. S4 and the other orbitals (HOMO−1/LUMO + 1 and HOMO−2/LUMO + 2) along with their respective energy gaps are presented in Fig. S5 in the supplementary information part.

**Table 1:** HOMO/LUMO energies and band gaps of entitled compounds.

Compounds	*E*_HOMO_	*E*_LUMO_	*E*_gap_
TPBR1	− 6.444	− 2.832	3.612
TPBD2	− 5.242	− 2.482	2.760
**TPBD3**	− 5.245	− 2.928	2.317
**TPBD4**	− 5.229	− 2.274	2.955
**TPBD5**	− 5.233	− 2.238	2.995
**TPBD6**	− 5.240	− 2.873	2.367

Units in eV; *E*_gap_ = *E*_LUMO_ − *E*_HOMO_.

The HOMO is depicted as the region with high electron donating ability i.e., donor, while the LUMO is found on an electron deficient part of molecule i.e., acceptor moiety and some portion of π-spacer^41^. The pictorial display is shown with red (negative) and blue (positive) colored regions to elaborate the electronic distribution patterns during the intramolecular charge transfer. A similar distribution pattern of HOMO and LUMO is seen in all the designed compounds (**TPBD2-TPBD6**) in contrast to **TPBR1** which shows a different pattern owing to the absence of a strong donor. The experimentally determined values of HOMO/LUMO (− 5.50/− 3.85 eV)^18^ in case of **TPBR1** show harmony with DFT values (− 6.444/− 2.832 eV) which indicated the suitable selection of functional for computational analysis. The lower band gaps in all the tailored compounds (2.995–2.317 eV) as compared to reference molecule (3.612 eV) defend their reactive nature with enhanced NLO effects^42^. The compound **TPBD5** showed the *E*_gap_ of 2.995 eV which is reduced owing to the presence of D–π–A framework that is absent in **TPBR1**. This energy gap is further reduced in **TPBD4** i.e., 2.955 eV which is attributed to the presence of a stronger DMF (Fig. S3) acceptor species in the side chain of molecule. Further reduction in the band gap is examined, such as 2.760 and 2.367 eV for **TPBD2** and **TPBD6**, respectively. In fact, the structure of **TPBD2** is fabricated via the replacement of two fluoro (–F) groups present in **TPBD4** with two chloro (–Cl) in the vicinity of an acceptor moiety i.e., DCF. Similarly, the compound **TPBD6** is designed with another acceptor named as NMF which has incorporated stronger electronegative –NO_2_ groups. As it is expected that inductive effect (-*I* effect) is directly related to the electronegativity of species which greatly influences the electronic charge transfer towards the acceptor moiety. The –F group is found to be more electronegative than –Cl group. Simultaneously, both –F and –Cl groups are electron donating due to resonance (–Cl > –F) and their inductive effect may compete with the resonance effect^43^. However, the reduction in the band gap of **TPBD2** and **TPBD6** may be due to (i) better orbital matching, (ii) resonance effect and (iii) particular geometry of the incorporated acceptor species (DCF and NMF, respectively). Among theoretically designed derivatives, we have predicted **TPBD3** with least band gap (2.317 eV) is regarded as the most suitable NLO material encompassing highly electron withdrawing acceptor DCN (Fig. S3). The band gap of **TPBD3** is found to be 1.559 times less than the *E*_gap_ of reference molecule. The reduction in energy gap is indicative to the combined effect of strong electronegative groups like nitro (–NO_2_) and chloro (–Cl) in the acceptor part of **TPBD3**. Thus, a strong electron push–pull force is generated in the molecule leading to fast electron transfer and rapid response as compared to the other derivatives. Hence, the decreasing order of HOMO–LUMO band gaps in the entitled compounds is as follows: **TPBR1** > **TPBD5** > **TPBD4** > **TPBD2** > **TPBD6** > **TPBD3**. Overall, a significant assistance of charge transference is observed in the designed derivatives as compared to the reference molecule which is a benchmark in their polarizable nature as proficient and reactive NLO materials.

### Global reactivity parameters (GRPs) analysis

The global reactivity descriptors such as electron affinity (*EA*), ionization potential (*IP*), electronegativity (*X*), global softness (*σ*), electrophilicity index (ω), global hardness (*η*) and chemical potential (*μ*) are calculated for **TPBR1** and **TPBD2-TPBD6** by utilizing the energies of FMOs (*E*_gap_ = *E*_LUMO _– *E*_HOMO_)^44,45^. Therefore, HOMOs and LUMOs are supplementary in forecasting the chemical nature of a molecule. The *IP* and *EA* are estimated by using the following Equations.

1 $$IP = - E_{{{\text{HOMO}}}}$$

2 $$EA = - E_{{{\text{LUMO}}}}$$

The chemical hardness^46^, chemical potential, electronegativity^47^, global softness and electrophilicity index^48^ are determined by employing the Koopman’s theorem^49,50^. The findings of global reactivity descriptors are computed and confined in Table 2.

3 $$X = \frac{{\left[ {IP + EA} \right]}}{2}$$

4 $$\eta = \frac{{\left[ {IP - EA} \right]}}{2}$$

5 $$\mu = \frac{{E_{{{\text{HOMO}}}} + E_{{{\text{LUMO}}}} }}{2}$$

6 $$\sigma = \frac{1}{2\eta }$$

7 $$\omega = \frac{{\mu^{2} }}{2\eta }$$

**Table 2:** The global reactivity descriptors of all the entitled compounds.

Comp	*IP*	*EA*	*X*	*η*	*μ*	*ω*	$\sigma$
TPBR1	6.444	2.832	4.638	1.806	− 4.638	5.955	0.277
TPBD2	5.242	2.482	3.862	1.380	− 3.862	5.404	0.362
**TPBD3**	5.245	2.928	4.087	1.159	− 4.087	7.207	0.432
**TPBD4**	5.229	2.274	3.752	1.478	− 3.752	4.763	0.338
**TPBD5**	5.233	2.238	3.736	1.498	− 3.736	4.659	0.334
**TPBD6**	5.240	2.873	4.057	1.184	− 4.057	6.952	0.422

Units in eV.

The ionization potential demonstrates the electron donating and accepting capabilities (the energy required to transmit an electron from HOMO). The *IP* values of derivatives are examined to be smaller than that of their parent chromophore which refer the easier releasing of electrons from designed chromophores (**TPBD2-TPBD6**) with smaller energy required to make them polarized than **TPBR1**. Interestingly, **TPBR1** manifested larger value of *EA* (2.832 eV) than quinoline–carbazole chromophores (1.07 eV)^51^ which supported the acceptor nature of this non-fullerene chromophore. Further, all the designed compounds expressed the comparable values of *EA* with **TPBR1** which elucidated their greater electron accepting nature. This might be due to the presence of robust electron-acceptor moieties (DCF, DCN, DMF, FMF and NMF, respectively). Greater values of electronegativity and electrophilicity index of entitled chromophores than quinoline–carbazole molecules also supported the aforesaid statement. The chemical potential ($\mu )$ is correlated to the reactivity and stability of the compounds^52^. The hardness, chemical potential and stability of a molecule have direct relation with the band gap while the reactivity has an inverse relationship. Therefore, a compound with larger energy gap is evaluated to be harder with more kinetic stability and least reactivity. However, the compound with smaller band gap is observed to be softer, highly reactive and less stable^53^. The overall decreasing order of softness values is: **TPBD3** > TBPD6 > TPBD2 > TBPD4 > **TPBD5** > TBPR1 which is exactly opposite to the hardness, chemical potential and band gap. A relatively smaller values of hardness (*η* = 1.159–1.498 eV) with relatively greater values of softness (*σ* = 0.334–0.432 eV) are seen in **TPBD2-TPBD6** as compared to **TPBR1** (*η* = 1.806 eV and *σ* = 0.227 eV). This might be because of vigorous acceptor units that made the derivatives more reactive as well as polarized and less stable which might attained probable NLO aptitude.

### UV–Vis analysis

In order to calculate the electronic excitation spectra, UV–Vis analysis for entitled chromophores was carried out in chloroform and gas phase employing TD-DFT computations^54^ using M062X/6-311G(d,p) functional. UV–Vis spectral analysis provides a useful insight of the nature of electronic transitions, probability of charge transfers and contributing configurations in all of the studied molecules^55,56^. Moreover, the study builds a relationship between the chemical structures of derivatives and their performance as efficient NLO material^57^. By performing TD-DFT computations, six lowest singlet–singlet allowed transitions are evaluated for **TPBR1** and **TPBD2-TPBD6** and results are confined in Tables S2–S13 while some major results are shown in Tables 3 and 4 and their absorption spectra in chloroform as well as in gaseous phase is shown in Fig. 3. Moreover, the influence of bridging core unit and acceptor moieties are investigated on the spectral properties of molecules under study.

**Table 3:** Computed absorption responses of **TPBR1** and **TPBD2-TPBD6** in chloroform solvent phase.

Compounds	DFT *λ*_max_ (nm)	E(eV)	*f*_os_	Major MO contributions (%)
TPBR1	568.214	2.182	2.392	H → L (78%)
TPBD2	542.862	2.284	1.224	H-1 → L (25%), H → L (65%)
**TPBD3**	571.145	2.171	1.466	H-3 → L (16%), H-1 → L (65%)
**TPBD4**	504.637	2.457	1.254	H-1 → L (28%), H → L (59%)
**TPBD5**	497.309	2.493	1.360	H-1 → L (31%), H → L (54%)
**TPBD6**	532.807	2.327	0.883	H-1 → L + 1(77%), H → L + 3(11%)

**Table 4:** Computed absorption responses of **TPBR1** and **TPBD2-TPBD6** in gas phase.

Compounds	*λ*_max_ (nm)	E(eV)	*f*_os_	Major MO contributions (%)
TPBR1	547.488	2.265	1.920	H → L (76%)
TPBD2	483.897	2.562	1.175	H-1 → L (59%), H → L (24%)
**TPBD3**	534.668	2.319	1.472	H-3 → L (13%), H-1 → L (70%)
**TPBD4**	458.844	2.702	0.991	H-1 → L (55%), H → L (26%)
**TPBD5**	460.326	2.693	1.018	H-1 → L (55%), H → L (25%)
**TPBD6**	527.211	2.352	1.408	H-3 → L (13%), H-1 → L (70%)

**Figure 4:**
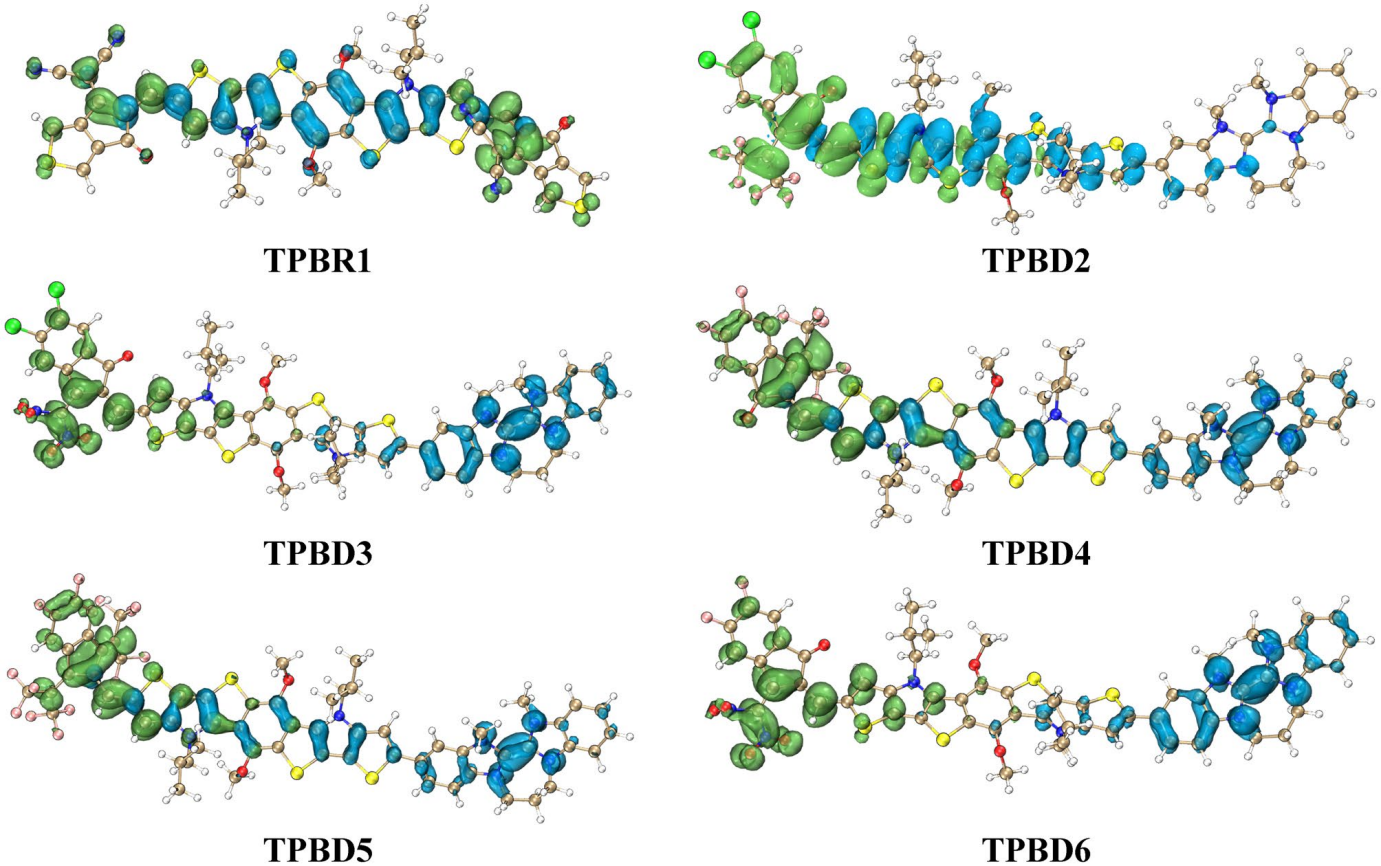
Hole + electron surface plot for excitation S_0_ → S_1_ of compound entitled chromophores. Hole is the blue surface and electron is the green surface. These surfaces are drawn by utilizing VMD software (https://www.ks.uiuc.edu/Research/vmd/) All out put files of entitled compounds were computed through Gaussian 09 version D.01 (https://gaussian.com/g09citation/).

It is expected that the polar medium elaborated that in π–π* state stabilization connected with the n–π* state is intended to be achieved by the use of appropriate electrical levels^58^. Usually, energy of interactions of a chromophore in chloroform solvent is explicated by the effects of polarity and non-covalent interactions (NCIs)^59^. Therefore, the dipole–dipole interactions and hydrogen bonding are significant in the stabilization of first singlet energy level of a molecule^60^. It has been generally observed that the solvent polarity induces a bathochromic shift in the absorption wavelength. Since, excited state is regarded more polar than the ground state and hence, more stabilizing the excited state as compared to ground state in chloroform^61^. The data from the above table exploited that **TPBR1** and **TPBD2-TPBD6** show absorbance in the UV–Visible region. For **TPBR1** the simulated *λ*_max_ is observed as 568.214 and 547.488 nm in chloroform and gas phase, respectively. Similarly, the calculated maximum absorbance values for **TPBD2-TPBD6** show greater bathochromic shift (571.145–497.309 nm) in polar solvent (chloroform) than that of gaseous phase (534.668–458.844 nm) as exhibited in Fig. 4. This might be because of the solvent effect as explained above.

Among **TPBD2-TPBD6** derivatives, it is apparent the highest value of *λ*_max_ is observed in **TPBD3** i.e. 571.145 and 534.668 nm with an oscillator strength (*f*_*os*_) of 1.466 and 1.472 in chloroform solvent and gas phase, respectively. Availability of strong electron withdrawing nitro (–NO_2_) and chloro (–Cl) groups on the acceptor region and extended conjugation might be the reason behind this red shift in **TPBD3**. Consequently, it has the smaller value of excitation energy (2.171 and 2.319 eV) as wavelength and excitation energy have inverse relation to each other. Conversely, **TPBD5** compound has shown the lowest *λ*_max_ of 497.309 nm along with the highest excitation energy and oscillator strength of 2.493 eV and 1.360, respectively in the chloroform solvent. While, in a gas phase, the minimum value of *λ*_max_ and maximum transition energy is observed for **TPBD4** (458.844 nm and 2.702 eV). A comparative study of maximum absorption wavelengths of all investigated compounds has revealed the following decreasing order: **TPBD3** > **TPBR1** > **TPBD2** > **TPBD6** > **TPBD4** > **TPBD5** in chloroform solvent and **TPBR1** > **TPBD3** > **TPBD6** > **TPBD2** > **TPBD5** > **TPBD4** in gas phase. The small drop in *λ*_max_ value stemmed from the replacement of toxic –CN group in the terminal regions of **TPBR1** with –CF_3_, –NO_2_, –F and –Cl in the derivatives posing environmental friendly compounds with less toxic impact^62^. Another crucial parameter to evaluate the performance of NLO materials is the excitation or transition energy^63^. It has been seen in literature that molecule having lower excitation energy possess greater charge transport ability. The presented TD-DFT calculations support our claim that the chromophores studied with the highest *λ*_max_ and lowest excitation energy values, appeared to be a suitable candidate for potential NLO applications.

### Hole-electron analysis

In the hole-electron analysis, an excited electron, in the hole region, leaves the hole to the electron region, is a hole-to electron excitation^64–66^. Data of hole-electron investigation is utilized to estimate the degree of separation and extent of distance between electronic distributions and hole (shown by *t* and *D* indices, respectively). Extent of overlap among hole and electron is calculated by S_r_ surface index, whereas, electron–hole distribution range is calculated by *H* index. The coulomb energy of attraction among the hole and electron, the delocalization index of hole and the delocalization index of electron is presented by E_Coul_, HDI and EDI, respectively^67,68^. In order to execute hole-electron investigation of **TPBR1** and **TPBD2-TPBD6** excitation possessing highest *f*_os_ (S_0_ → S_1_ excitations) are used and their indices are exhibited in Table 5 while hole + electron surfaces are presented in the Fig. 4.

**Table 5:** Hole-electron analysis indices for S_0_ → S_1_ excitation (with highest *f*_osc_) of compounds.

Compounds	TPBR1	TPBD2	**TPBD3**	**TPBD4**	**TPBD5**	**TPBD6**
Excitation	S_0_ → S_1_	S_0_ → S_1_	S_0_ → S_1_	S_0_ → S_1_	S_0_ → S_1_	S_0_ → S_1_
*λ* (nm)	568.21	542.86	571.14	504.63	497.30	532.80
*f*_os_	2.39	1.22	1.46	1.25	1.36	0.88
E (eV)	2.18	2.28	1.92	2.457	2.49	1.97
*D* (Å)	0.85	15.08	20.83	13.33	12.82	20.69
E_Coul_ (eV)	2.44	1.37	0.73	1.55	1.60	0.73
S_r_ (a.u.)	0.56	0.36	0.15	0.42	0.43	0.16
*H* (Å)	7.55	6.462	4.77	6.686	6.789	4.81
*t* (Å)	− 3.08	8.92	16.51	6.966	6.346	16.30
HDI	4.39	4.64	6.35	4.48	4.32	6.34
EDI	4.15	5.78	6.69	5.35	5.07	6.67

**Figure 5:**
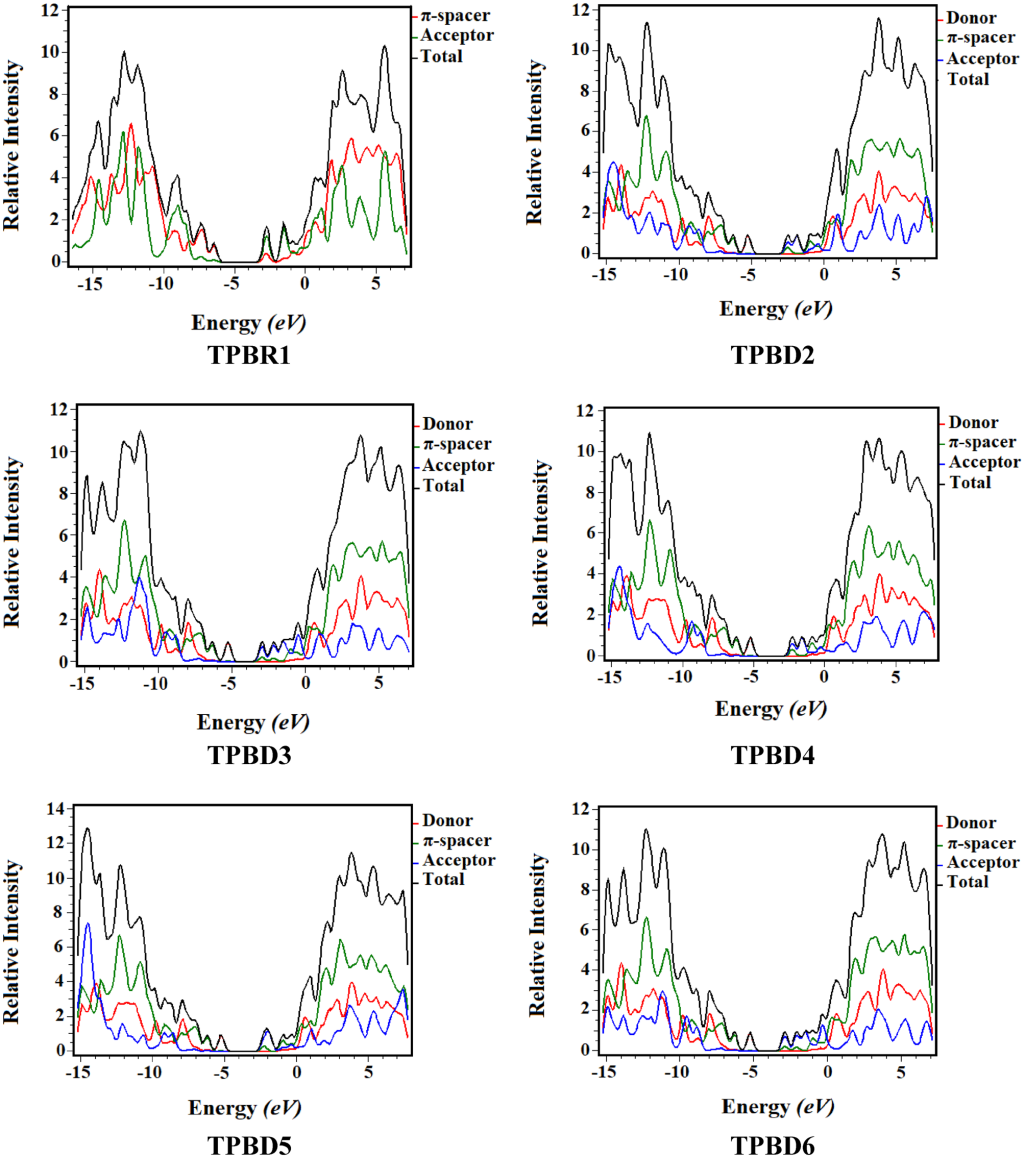
A pictorial display of DOS for **TPBR1** and **TPBD2-TPBD6**. Figures are drawn by utilizing PyMOlyze 1.1 version (https://sourceforge.net/projects/pymolyze/). All out put flies of entitled compounds were computed through Gaussian 09 version D.01 (https://gaussian.com/g09citation/).

The hole-electron investigation report S_0_ → S_1_ excitation in **TPBD3** needs less energy i.e. 1.92 eV demonstrating easily excitations favored by –NO_2_ and –Cl groups relative to **TPBR1**, **TPBD2**, **TPBD4**, **TPBD5** and **TPBD6** possessing energies of 2.18, 2.28, 2.46, 2.49 and 1.97 eV, respectively. Interestingly, in all derivatives for S_0_ → S_1_ excitation, a huge separation among distributions of hole and electron than that of reference compound as these chromophores exhibited the high *D* and *t* index values (see Table 5). Further the distribution surface plot of holes and electron also confirmed their hole and electron distribution in different regions as shown in Fig. 5. This separation in turn shows the huge charge transference (CT) in derivatives for S_0_ → S_1_ excitation as compared to **TPBR1**. As S_0_ → S_1_ transition, exhibit low excitation (LE) owing to their less separation among distributions of hole and electron (shown in Fig. 4), which is further validated by the least value of *D* index i.e. and *t* index*.* 0.85 Å and t < 0 (− 3.08 Å), respectively indicating hole and electron distribution in the same region in **TPBR1**. The higher S_r_ indices values (0.16–0.56 a.u) in entitled chromophores for S_0_ → S_1_ excitations indicates π–π ∗ transitions in these molecules. Consequently, among all studied compounds, **TPBD3** with highest HDI (6.35) and EDI (6.69), proves upon substituting –NO_2_ and –Cl groups enhances electronic delocalization favoring non-covalent interactions.

### Density of states (DOS)

The density of states (DOS) analysis is carried out to support the results of **TPBR1** and **TPBD2-TPBD6**, accompanied by FMO observation^16^. To perform DOS investigations, the studied compounds are fragmented as donor, π-core and end-capped acceptor unit each represented by a different color (D with red, π-spacer with green and A with blue lines) as indicated in Fig. 5. The pattern of charge distribution is altered by changing acceptor moieties and is defended by the HOMO–LUMO percentages of DOS. Herein, donor displays charge contribution pattern as: 96.8, 96.9, 96.5, 96.5 and 96.5% to HOMO whereas, 0.2, 0.1, 0.2, 0.2 and 0.2% to LUMO for **TPBD2-TPBD6**, respectively. Similarly, π-linker shows contribution of charge as: 87.5, 3.2, 3.1, 3.4, 3.5 and 3.5% to HOMO while, 29.1, 36.4, 23.4, 34.6, 32.6 and 32.6% to LUMO for **TPBR1** and **TPBD2-TPBD6**, accordingly. Likewise, acceptor manifests distribution of electronic charge as: 12.5, 0.1, 0.1, 0.0, 0.0 and 0.0% to HOMO whereas, 70.9, 63.4, 76.5, 65.1, 67.1 and 67.1% to LUMO for **TPBR1** and **TPBD2-TPBD6**, correspondingly. In DOS pictographs, the valence band (HOMO) showed the negative values and the conduction band (LUMO) displayed the positive values along x-axis and energy gap is demonstrated by the distance between them^32^. In case of **TPBR1**, the charge density for HOMO entirely lies on π-spacer (87.5% contribution) and for LUMO it occurs on terminal acceptors (79% acceptor contribution for LUMO). However, in **TPBD2-TPBD6**, the relative density for HOMO completely exists on donor (96.8, 96.9, 96.5, 96.5 and 96.5% donor contribution to HOMO in **TPBD2-TPBD6**, respectively while for LUMO it is majorly occupied by acceptor and to some extent by π-linker. Overall pattern of charge distribution elucidated that electron delocalization is perceived and an appreciable amount of charge is shifted from peripheral donor to end-capped acceptor through a π-bridge in **TPBR1** and **TPBD2-TPBD6**.

### Non-linear optical (NLO) study

**Figure 6:**
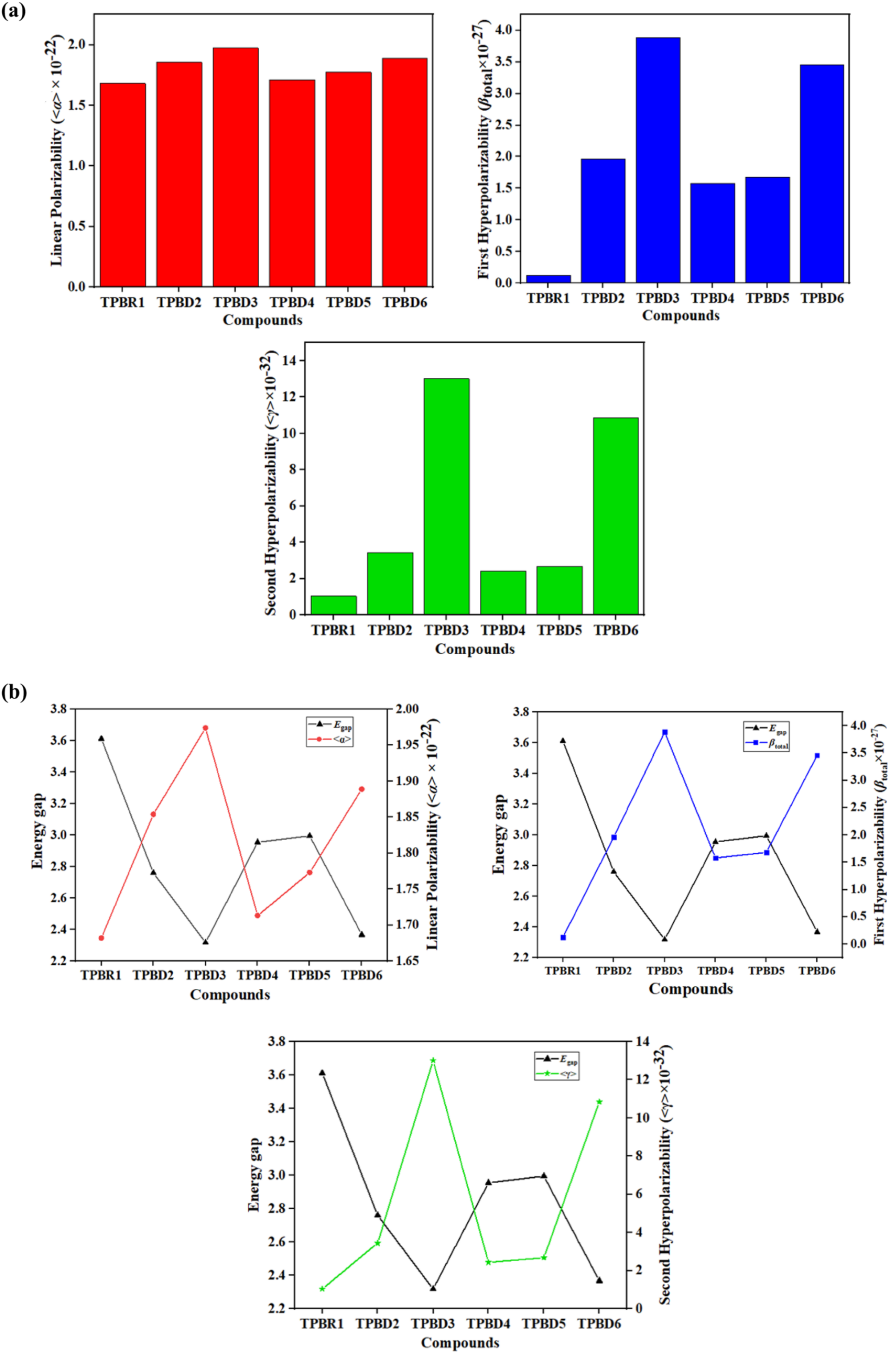
(**a**) Graphical representation of linear polarizability and hyperpolarizabilities of entitled compounds. These graphs are drawn by utilizing Origin Pro 8.5 version (https://originpro.informer.com/8.5/). All out put flies of entitled compounds were computed through Gaussian 09 version D.01 (https://gaussian.com/g09citation/). (**b**) A comparison of <a> , *β*_total_, $\gamma$_total_ with energy band gap (*E*_HOMO − LUMO_) of **TPBR1** and **TPBD2-TPBD6**. These graphs are drawn by utilizing Origin Pro 8.5 version (https://originpro.informer.com/8.5/). All out put flies of entitled compounds were computed through Gaussian 09 version D.01 (https://gaussian.com/g09citation/).

The NLO investigations have been recognized as the most promising technology towards the advancement of several fields such as photonics, optoelectronics and biomedicine^69^. For generating the NLO response, pull–push architecture of compounds is established whose strength depends upon the nature of D and A moieties that are inter-linked through π-framework^58^. The various NLO parameters include linear polarizability <a> , first (*β*_total_) and second ($\gamma$_total_) hyperpolarizabilities of **TPBR1** and **TPBD2-TPBD6** are studied in gas phase and promising results are obtained which are expressed in Tables S14–S17. The Fig. 6a in manuscript displayed a graphical representation of <a> , *β*_total_ and $\gamma$_total_ for the studied compounds while, the Fig. 6b shows their comparison with energy gap values. The average polarizability <a> ^70^ value is estimated with the help of Eq. (8).

8 $$< a > = {1}/{3 }(\alpha_{{{\text{xx}}}} + \, \alpha_{yy} + \, \alpha_{zz} )$$

The first (*β*_total_)^71^ and second ($\gamma$_total_)^72^ hyperpolarizabilities are calculated using the Eqs. (9) and (10).

9 $$\beta_{{{\text{total}}}} = \left( {\beta_{(x)}^{2} + \beta_{y}^{2} + \beta_{z}^{2} } \right)^{1/2}$$

where *β*_x_ = *β*_xxx+_*β*_xyy+_*β*_xzz_, *β*_y_ = *β*_yxx+_*β*_yyy+_*β*_yzz_ and *β*_z_ = *β*_zxx+_*β*_zyy+_*β*_zzz_.

10 $$\gamma_{{{\text{total}}}} = \sqrt {{\upgamma }_{x }^{2} + {\upgamma }_{y}^{2} + {\upgamma }_{z}^{2} }$$

where $${\gamma }_{i}= \frac{1}{15 }\sum_{j}({\gamma }_{ijji}+{\gamma }_{ijij}+{\gamma }_{iijj})\;i,j = \{x, y, z\}$$

The dipole moment is estimated by the help of Eq. (11).

11 $$\mu = \, \left( {\mu^{{2}}_{x} + \mu^{2}_{y} + \mu^{{2}}_{z} } \right)^{{{1}/{2}}}$$

The dipole moment (*u*) is an important factor to estimate polarizability of organic chromophores^73^. It is the product of charge magnitudes and distance between them. Table S15 pointed out the total estimated values of dipole moment (*u*_total_) along with the three-dimensional tensors along x, y and z-axis. All the designed chromophores expressed greater dipole moment (9.854–16.686 D) than that of their parent chromophore (9.208 D). The greatest value of *u*_total_ is found in **TPBD3** and **TPBD6** i.e.16.686 and 16.476 *D*, respectively because of the presence of strong acceptor units (DCF and NMF, respectively). The findings in the literature revealed that urea was utilized as a standard chromophore for comparative analysis in NLO study and dipole moment for urea is reported as 1.3732 D^74^. The *u*_total_ values of **TPBR1**, **TPBD2**, **TPBD3**, **TPBD4**, **TPBD5** and **TPBD6** are 6.71, 9.55, 12.15, 7.18, 7.58 and 11.99 times greater than urea. The higher values of *u*_total_ for the entitled chromophores exploited the higher polarizability in them. In all studied chromophores, the greater dipole moment values are examined along *u*_x_ (**TPBR1** = 7.640, **TPBD2** = 13.085, **TPBD3** = 16.447 and **TPBD6** = 16.295 D) except **TPBD4** and **TPBD5** which exhibited higher values along *u*_z_ (3.160 and 3.432 D, respectively). Further, linear polarizability behavior of entitled chromophores is also studied and all the derivatives showed comparable values of the <a> with **TPBR1** and comparison between values is shown in Fig. 6a. The date in Table S14 explained that all the entitled chromophores exhibited greater responses of <a> along x-axis (*α*_*xx*_). Overall the <*a*> values of the studied chromophores decrease in the following order: **TPBD3** (1.974 × 10^–22^) > **TPBD6** (1.889 × 10^–22^) > **TPBD2** (1.854 × 10^–22^) > **TPBD5** (1.773 × 10^–22^) > **TPBD4** (1.713 × 10^–22^) > **TPBR1** (1.682 × 10^–22^ esu).

In fullerene free chromophores, the NLO properties can be recognized from the charge transfer efficiency towards A moiety from D unit through their respective π-bridges. Concisely, enhancement in hyperpolarizability values in **TPBD2-TPBD6** rise in alliance with the delocalization of π-electrons. This delocalization reduces the HOMO/LUMO energy gap. As explained in the literature, polarizability of chromophores is greatly affected by the HOMO–LUMO band gap i.e., the smaller the band gap, the larger will be the polarizability values and vice versa^45^. The same trend is examined for chromophores in our studied case where compound **TPBD3** showed the greatest *β*_*total*_ value i.e. 3.885 × 10^–27^ esu with lowest band gap i.e. 2.317 eV. A comparison between *β*_total_ values and energy gap between HOMO/LUMO orbitals is shown in Fig. 6b. The *β*_total_ value of **TPBR1** is 0.122 × 10^–27^ esu and all tailored chromophores expressed significant results (1.578–3.885 × 10^–27^ esu) than that of **TPBR1** because of the strong push–pull configuration. Additionally, a systematical relationship is seen between the molecular structures and *β*_total_ values. The *β*_total_ parameter usually enhanced with the extended conjugated system and strength of the “A” substituents attached like –Cl, –F and –NO_2_ groups that are contributing to chromophore nonlinearity. In accordance to the above mentioned results, the highest *β*_total_ is obtained for **TPBD3** i.e., 3.885 × 10^–27^ esu. The reason might be hidden in the well-defined D and A moieties utilized in the structure of **TPBD3** (Fig. S1). A decreasing trend of *β*_total_ values of all the aforementioned compounds is as follows: **TPBD3** (3.885 × 10^–27^) > **TPBD6** (3.460 × 10^–27^) > **TPBD2** (1.963 × 10^–27^) > **TPBD5** (1.681 × 10^–27^) > **TPBD4** (1.578 × 10^–27^) > **TPBR1** (0.122 × 10^–27^ esu). The contributing tensors of *β*_total_ (*β*_xxx_, *β*_xxy_, *β*_xyy_ etc.) also play a significant role in determining the overall *β*_total_ values. From the profound observation, it is estimated that in compounds **TPBR1**, **TPBD2**, **TPBD3** and **TPBD6**, the *β*_xxx_ (x-axis) is majorly contributing in the *β*_total_ as indicated by their positive high values of 0.020, 1.969, 3.894 and 3.467 × 10^–27^ esu, respectively. In the remaining compounds, y-axis also contributes in addition to horizontal axis. The compounds **TPBD4** and **TPBD5** show a clear contribution of x and y-axis from their *β*_xxy_ contributing tensor values as 3.383 and 7.499 × 10^–29^ esu, respectively. A comparative analysis is also performed using urea as standard molecule (*β*_total_ = 0.372 × 10^–30^ esu)^74^. The second-order hyperpolarizabilities computed for **TPBR1**, **TPBD2**, **TPBD3**, **TPBD4**, **TPBD5** and **TPBD6** are 327.95, 5276.88, 10,443.55, 4241.94, 4518.82 and 9301.08 times higher than that of urea. This comparative study with urea exhibited that entitled chromophores showed excellent NLO responses. The results of third-order NLO parameter ($\gamma$_total_) are confined at the end are also in our support showing the utmost value of $\gamma$_total_ in case of **TPBD3** as 13.02 × 10^–32^ esu. The $\gamma$_total_ values decrease in the following order: **TPBD3** (13.02 × 10^–32^) > **TPBD6** (10.85 × 10^–32^) > **TPBD2** (3.439 × 10^–32^) > **TPBD5** (2.677 × 10^–32^) > **TPBD4** (2.424 × 10^–32^) > **TPBR1** (1.039 × 10^–32^ esu). Generally, it has been observed that all the derivatives hold polarizable nature and are obtained with less band gap than **TPBR1**. In particular, compound **TPBD3** has secured the highest position amidst all NLO candidates on account of its highest <a> , *β*_total_ and $\gamma$_total_ findings and is anticipated as emerging NLO substance in the technology related applications.

## Conclusion

Herein, a series of chromophores (**TPBD2-TPBD6**) is designed with strong push–pull configurations from **TPBR1** via modification with various acceptor moieties. Due to the change in configuration from A–π–A (**TPBR1**) to D–π–A (**TPBD2-TPBD6**), a marvelous NLO response is examined. FMO analysis showed that an effective transfer of charge has taken place from donor to acceptor through π-bridge in all derivatives with reduction in the band gap (2.317–2.995 eV) than **TPBR1** (3.612 eV). Moreover, the GRP studies revealed that conjugation in chromophores causes the exceptional stability in the studied organic molecules. The greater dipole moment (*u*_total_ = 9.854–16.686 D) are found for derivatives than parent chromophore which predicts that larger polarizability results in attractive NLO responses. An outstanding NLO findings: <a> , *β*_total_ and $\gamma$_total_ values are noted to be 1.974 × 10^–22^, 3.885 × 10^–27^ and 13.02 × 10^–32^ esu, respectively for **TPBD3**, which are highest among all said chromophores. Hence, we can conscript the reasonable aptitudes of our derivatives for better NLO properties that recommended their utilization in high-tech photonic appliances.

## Supplementary Information

Supplementary Information.

## Data Availability

All data generated or analyzed during this study are included in this published article and its supplementary information files.
