# Supplementary material for: Theoretical designing of non-fullerene derived organic heterocyclic compounds with enhanced nonlinear optical amplitude: a DFT based prediction
Source: Sci Rep. 2022 Nov 23;12:20220. doi: 10.1038/s41598-022-21894-x (PMC9684159; doi:10.1038/s41598-022-21894-x)
Supplement: Supplementary file 1 — Supplementary Information. [file 41598_2022_21894_MOESM1_ESM.docx]

**Supplementary Information**

**Theoretical Designing of Non-Fullerene Derived Organic Heterocyclic Compounds with Enhanced Nonlinear Optical Amplitude: A DFT Based Prediction**

Muhammad Khalid*^1,2^, Mashal Khan^1,2^, Khalid Mahmood^3^, Muhammad Arshad^4^, Muhammad Imran^5^, Ataualpa Albert Carmo Braga^6^, Riaz Hussain^7^

^1^ Institute of Chemistry, Khwaja Fareed University of Engineering & Information Technology, Rahim Yar Khan, 64200, Pakistan

^2^Centre for Theoretical and Computational Research, Khwaja Fareed University of Engineering & Information Technology, Rahim Yar Khan, 64200, Pakistan

^3^Institute of Chemical Sciences, Bahauddin Zakariya University, Multan 60800, Pakistan

^4^Department of Chemical Engineering, College of Engineering, King Khalid University, Abha, Saudi Arabia.

^5^Department of Chemistry, Faculty of Science, King Khalid University, P.O. Box 9004, Abha 61413, Saudi Arabia.

^6^Departamento de Química Fundamental, Instituto de Química, Universidade de São Paulo, Av. Prof. Lineu Prestes, 748, São Paulo, 05508-000, Brazil.

^7^Department of Chemistry, Division of Science and Technology, University of Education Lahore, Pakistan

*Corresponding author E-mail addresses:

Dr. Muhammad Khalid (muhammad.khalid@kfueit.edu.pk; [Khalid@iq.usp.br](mailto:Khalid@iq.usp.br))

|  |  |
| --- | --- |
| **TPBR1** | **TPBD2** |
|  |  |
| **TPBD3** | **TPBD4** |
| **** | **** |
| **TPBD5** | **TPBD6** |


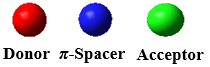


**Figure S1:** Structure of reference (**TPBR1**) and designed compounds (**TPBD2-TPBD6**).

| 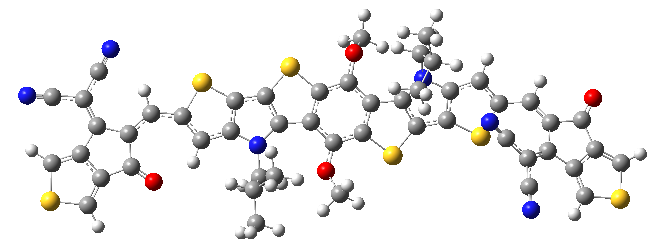 | 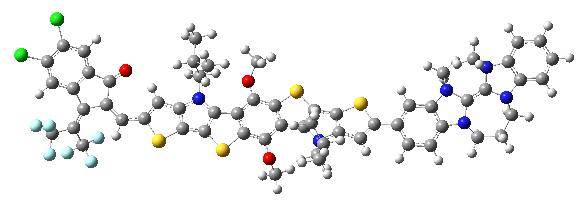 |
| --- | --- |
| **TPBR1** | **TPBD2** |
| 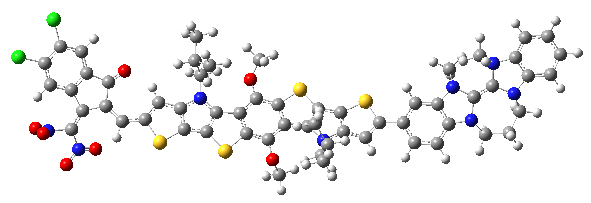 | 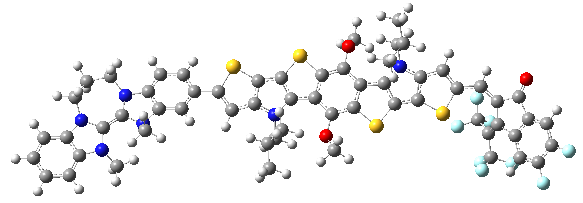 |
| **TPBD3** | **TPBD4** |
| 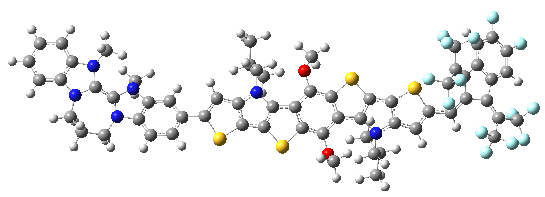 | 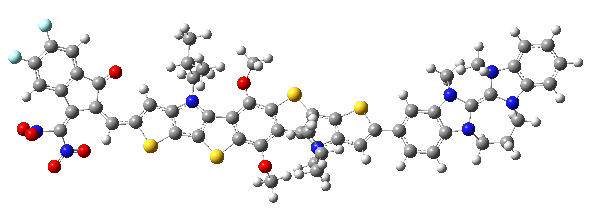 |
| **TPBD5** | **TPBD6** |


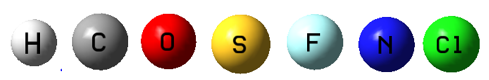


**Figure S2:** Optimized structures of reference compound (**TPBR1**) and designed compounds (**TPBD2-TPBD6**).

|   5,6-dichloro-2-methylene-3-(perfluoropropan-2-ylidene)-2,3-dihydro-1H-inden-1-one (**DCF**) |   5,6-dichloro-3-(dinitromethylene)-2-methylene-2,3-dihydro-1H-inden-1-one (**DCN**) |   5,6-difluoro-2-methylene-3-(perfluoropropan-2-ylidene)-2,3-dihydro-1H-inden-1-one (**DMF**) |   5,6-difluoro-2-methylene-1,3-bis(perfluoropropan-2-ylidene)-2,3-dihydro-1H-indene (**FMF**) | 3-(dinitromethylene)-5,6-difluoro-2-methylene-2,3-dihydro-1H-inden-1-one (**NMF**) |
| --- | --- | --- | --- | --- |

**Figure S3:** The structures of various acceptors with IUPAC names used in the designed compounds.

| **HOMO** | **LUMO** |
| --- | --- |
| **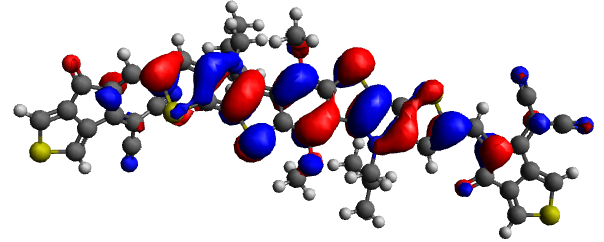** | **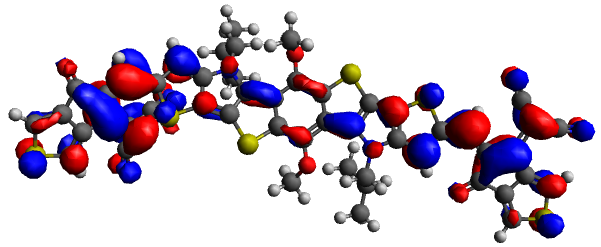** |
| **TPBR1** | |
| **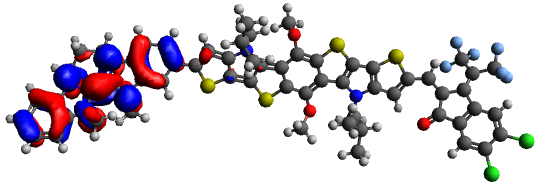** | **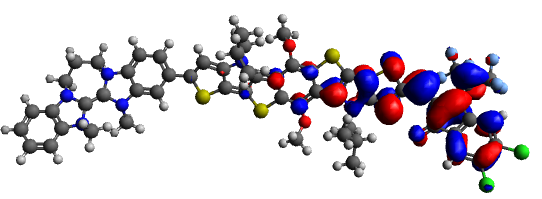** |
| **TPBD2** | |
| **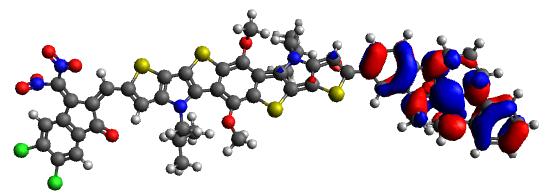** | **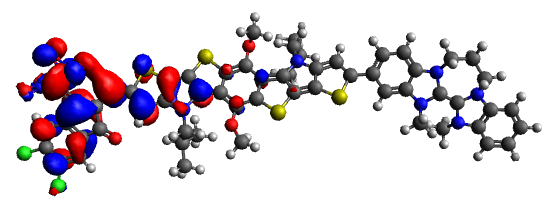** |
| **TPBD3** | |
| **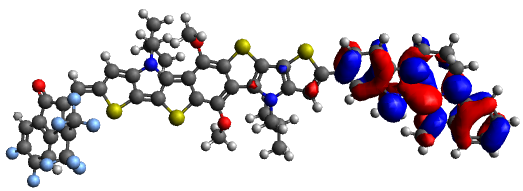** | **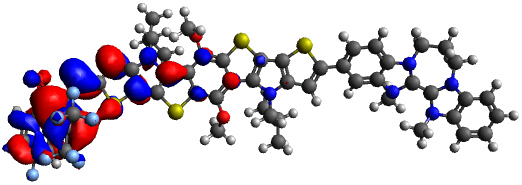** |
| **TPBD4** | |
| **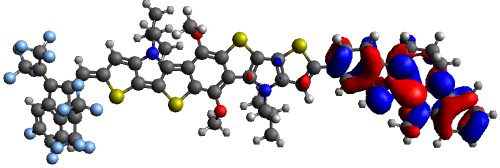** | **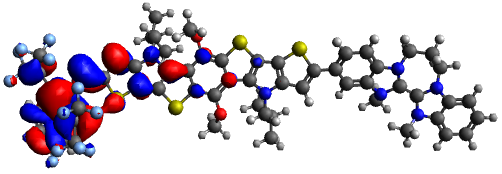** |
| **TPBD5** | |
| **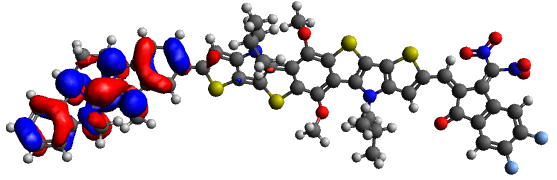** | **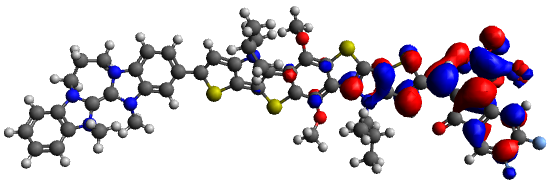** |
| **TPBD6** | |

**Figure S4:** HOMOs and LUMOs of the studied molecules (**TPBR1** and **TPBD2**-**TPBD6**).

| 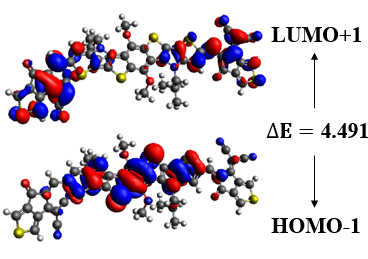 | 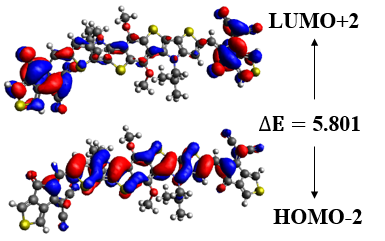 |
| --- | --- |
| **TPBR1** | |
| 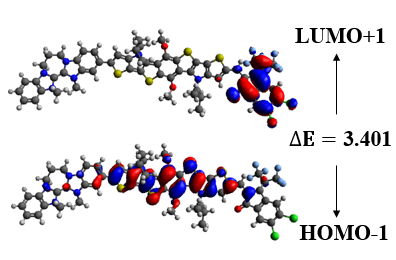 | 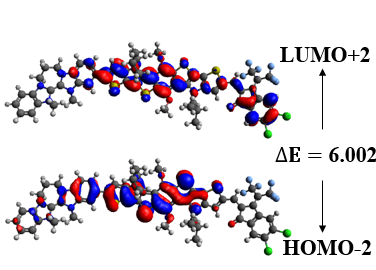 |
| **TPBD2** | |
| 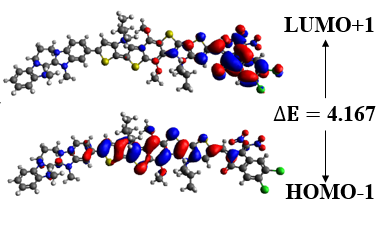 | 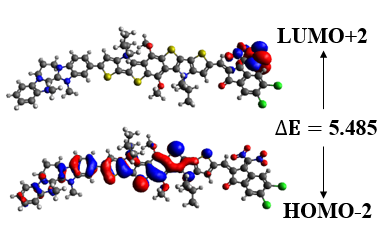 |
| **TPBD3** | |
| **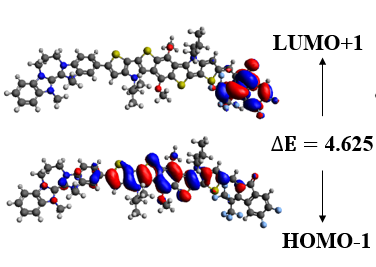** | **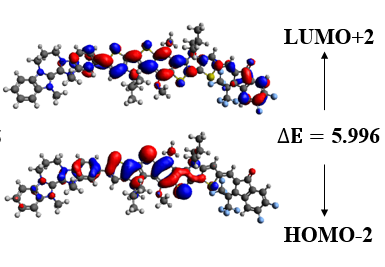** |
| **TPBD4** | |
| **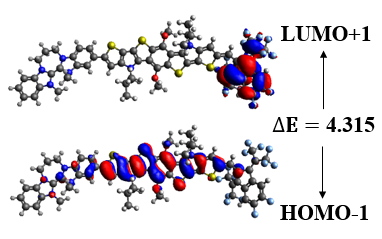** | **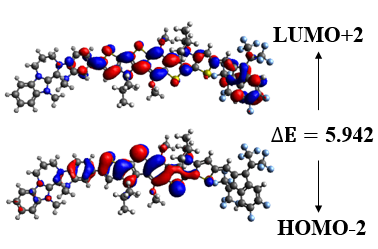** |
| **TPBD5** | |
| **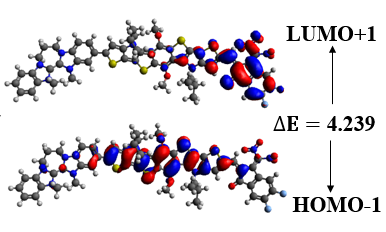** | **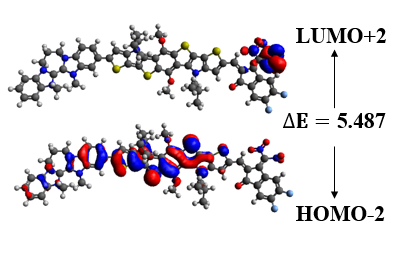** |
| **TPBD6** | |

**Figure S5:** Energy gap of HOMO-1/LUMO+1 and HOMO-2/LUMO+2 of the studied molecules **TPBR1** and **TPBD2**-**TPBD6**.

**Table S1:** Calculated energy (E) and energy gap ($\boldsymbol{\Delta}$E) of entitled compounds in *eV.*

| **TPBR1** | | | **TPBD2** | | **TPBD3** | |
| --- | --- | --- | --- | --- | --- | --- |
| **MO(s)** | **E (*eV*)** | $\boldsymbol{\Delta}$**E (*eV*)** | **E (*eV*)** | $\boldsymbol{\Delta}$**E (*eV*)** | **E (*eV*)** | $\boldsymbol{\Delta}$**E (*eV*)** |
| LUMO | -2.832 |  | -2.482 |  | -2.928 |  |
| HOMO | -6.444 | 3.612 | -5.242 | 2.760 | -5.245 | 2.317 |
| LUMO+1 | -2.635 |  | -1.841 |  | -2.148 |  |
| HOMO-1 | -7.126 | 4.491 | -5.242 | 3.401 | -6.315 | 4.167 |
| LUMO+2 | -1.550 |  | -0.934 |  | -1.485 |  |
| HOMO-2 | -7.351 | 5.801 | -6.936 | 6.002 | -6.970 | 5.485 |

**Table S1** (Continue) Calculated energy (E) and energy gap ($\boldsymbol{\Delta}$E) of entitled compounds in *eV.*

| **TPBD4** | | | **TPBD5** | | **TPBD6** | |
| --- | --- | --- | --- | --- | --- | --- |
| **MO(s)** | **E(*eV*)** | $\boldsymbol{\Delta}$**E (*eV*)** | **E (*eV*)** | $\boldsymbol{\Delta}$**E (*eV*)** | **E (*eV*)** | $\boldsymbol{\Delta}$**E (*eV*)** |
| LUMO | -2.274 |  | -2.238 |  | -2.873 |  |
| HOMO | -5.229 | 2.955 | -5.233 | 2.995 | -5.240 | 2.367 |
| LUMO+1 | -1.582 |  | -1.884 |  | -2.065 |  |
| HOMO-1 | -6.207 | 4.625 | -6.199 | 4.315 | -6.304 | 4.239 |
| LUMO+2 | -0.887 |  | -0.938 |  | -1.475 |  |
| HOMO-2 | -6.883 | 5.996 | -6.880 | 5.942 | -6.962 | 5.487 |

*E*= energy, $\Delta$*E*= *E*_LUMO_-*E*_HOMO;_ HOMO = highest occupied molecular orbital; LUMO = lowest unoccupied molecular orbital, MO= molecular orbital, comp=compounds.

**Table S2:** Wave length, excitation energy and oscillator strength of investigated compound **TPBR1** in chloroform solvent at M06-2X/6-311 G(d,p) level.

| **No.** | **DFT λ (*nm*)** | **E(*eV*)** | ***f*_os_** | **MO contributions (%)** |
| --- | --- | --- | --- | --- |
| 1 | 568.214 | 2.182 | 2.392 | H-2→L+1 (12%), H→L (78%), H-1→L (3%) |
| 2 | 502.938 | 2.465 | 0.178 | H-2→L (21%), H→L+1 (68%), H-3→L (2%), H-1→L+1 (3%) |
| 3 | 431.339 | 2.874 | 0.191 | H-3→L+1 (18%), H-1→L (68%), H→L (6%) |
| 4 | 414.982 | 2.988 | 0.097 | H-3→L (37%), H-1→L+1 (47%), H-4→L+1 (3%),H→L+1 (7%) |
| 5 | 366.644 | 3.382 | 0.012 | H-2→L (59%), H→L+1 (19%), H-4→L+1 (5%), H-3→L (2%), H-2→L+1 (4%) |
| 6 | 355.724 | 3.485 | 0.047 | H-4→L (17%), H-2→L+1 (39%), H→L+2 (13%), H-3→L+1 (6%), H-2→L (4%), H→L (5%), H→L+1 (3%), H→L+4 (3%) |

**Table S3:** Wave length, excitation energy and oscillator strength of investigated compound **TPBD2** in chloroform solvent at M06-2X/6-311 G(d,p) level.

| **No.** | **DFT λ (*nm*)** | | **E(*eV*)** | | ***f*_os_** | | **MO contributions (%)** | |
| --- | --- | --- | --- | --- | --- | --- | --- | --- |
| 1 | | 542.862 | | 2.284 | | 1.224 | | H-1→L (25%), H→L (65%), H-3→L (4%) |
| 2 | | 501.859 | | 2.471 | | 1.027 | | H-3→L (12%), H-1→L (50%), H→L (33%) |
| 3 | | 408.878 | | 3.032 | | 0.095 | | H→L+1 (79%), H-1→L+1 (5%), H→L+2 (7%), H→L+3 (2%) |
| 4 | | 402.873 | | 3.078 | | 0.361 | | H-4→L (25%), H-3→L (13%), H-2→L (45%), H-8→L (2%), H-5→L (4%) |
| 5 | | 392.045 | | 3.163 | | 0.399 | | H→L+1 (11%), H→L+2 (46%), H→L+3 (16%), H→L+4 (10%), H-3→L (3%), H-1→L+2 (3%) |
| 6 | | 381.255 | | 3.252 | | 0.059 | | H-3→L+1 (22%), H-1→L+1 (52%), H-10→L+1 (3%), H-5→L+1 (3%), H→L+1 (9%) |

**Table S4:** Wave length, excitation energy and oscillator strength of investigated compound **TPBD3** in chloroform solvent at M06-2X/6-311 G(d,p) level.

| **No.** | **DFT λ (*nm*)** | | **E(*eV*)** | | ***f*_os_** | | **MO contributions (%)** | |
| --- | --- | --- | --- | --- | --- | --- | --- | --- |
| 1 | | 645.516 | | 1.921 | | 0.409 | | H→L (90%), H-1→L (7%) |
| 2 | | 571.145 | | 2.171 | | 1.466 | | H-3→L (16%), H-1→L (65%), H-1→L+1 (3%), H→L (9%) |
| 3 | | 460.805 | | 2.691 | | 0.140 | | H→L+1 (85%), H-1→L+1 (6%), H→L+3 (3%) |
| 4 | | 441.712 | | 2.807 | | 0.115 | | H-4→L (20%), H-3→L (16%), H-2→L (34%), H-8→L (2%), H-5→L (7%), H-2→L+1 (4%), H-1→L+1 (6%) |
| 5 | | 419.319 | | 2.957 | | 0.577 | | H-3→L+1 (18%), H-1→L+1 (52%), H-5→L+1 (3%), H-4→L (2%), H-2→L (6%), H-1→L (2%), H→L+1 (8%) |
| 6 | | 399.125 | | 3.106 | | 0.237 | | H-3→L (22%), H→L+3 (23%), H→L+4 (11%), H-3→L+1 (3%), H-2→L (6%), H-1→L (9%), H-1→L+3 (3%), H→L+1 (3%), H→L+6 (5%) |

**Table S5:** Wave length, excitation energy and oscillator strength of investigated compound **TPBD4** in chloroform solvent at M06-2X/6-311 G(d,p) level.

| **No.** | **DFT λ (*nm*)** | **E(*eV*)** | ***f*_os_** | **MO contributions (%)** |
| --- | --- | --- | --- | --- |
| 1 | 504.637 | 2.457 | 1.254 | H-1→L (28%), H→L (59%), H-3→L (6%), H→L+2 (2%) |
| 2 | 466.720 | 2.657 | 0.795 | H-3→L (13%), H-1→L (44%), H→L (38%), |
| 3 | 395.421 | 3.136 | 0.560 | H→L+1 (12%), H→L+2 (49%), H→L+3 (16%), H-3→L (2%), H-1→L+2 (4%), H→L (3%), H→L+4 (7%) |
| 4 | 384.614 | 3.224 | 0.056 | H-4→L (28%), H-3→L (10%), H-2→L (40%), H-8→L (3%), H-5→L (4%), H-1→L (5%) |
| 5 | 374.111 | 3.314 | 0.021 | H→L+1 (80%), H-1→L+1 (4%), H→L+2 (8%), H→L+3 (3%) |
| 6 | 354.089 | 3.502 | 0.223 | H-3→L+1 (15%), H-1→L+1 (46%), H-4→L (2%), H-3→L (6%), H-2→L (3%), H-1→L+2 (8%), H→L+1 (7%) |

**Table S6:** Wave length, excitation energy and oscillator strength of investigated compound **TPBD5** in chloroform solvent at M06-2X/6-311 G(d,p) level.

| **No.** | **DFT λ (*nm*)** | **E(*eV*)** | ***f*_os_** | **MO contributions (%)** |
| --- | --- | --- | --- | --- |
| 1 | 497.309 | 2.493 | 1.360 | H-1→L (31%), H→L (54%), H-3→L (7%), H→L+2 (2%) |
| 2 | 461.045 | 2.689 | 0.689 | H-3→L (13%), H-1→L (40%), H→L (42%), |
| 3 | 413.763 | 2.997 | 0.018 | H→L+1 (91%), H-1→L+1 (6%) |
| 4 | 393.326 | 3.152 | 0.602 | H→L+2 (51%), H→L+3 (21%), H-3→L (2%), H-1→L+2 (5%), H→L (3%), H→L+4 (9%) |
| 5 | 385.307 | 3.218 | 0.012 | H-3→L+1 (23%), H-1→L+1 (54%), H-5→L+1 (3%), H→L+1 (8%) |
| 6 | 377.943 | 3.281 | 0.069 | H-4→L (25%), H-3→L (12%), H-2→L (39%), H-8→L (2%), H-5→L (4%), H-2→L+2 (2%), H-1→L (5%), H-1→L+1 (3%) |

**Table S7:** Wave length, excitation energy and oscillator strength of investigated compound **TPBD6** in chloroform solvent at M06-2X/6-311 G(d,p) level.

| **No.** | **DFT λ (*nm*)** | | **E(*eV*)** | | ***f*_os_** | | **MO contributions (%)** | |
| --- | --- | --- | --- | --- | --- | --- | --- | --- |
| 1 | | 1553.297 | | 0.798 | | 0.068 | | H→L (100%) |
| 2 | | 817.784 | | 1.516 | | 0.074 | | H→L+1 (99%) |
| 3 | | 749.602 | | 1.654 | | 0.000 | | H→L+2 (100%) |
| 4 | | 735.157 | | 1.687 | | 0.879 | | H-1→L (95%) |
| 5 | | 557.282 | | 2.225 | | 0.101 | | H-2→L (77%), H-5→L (3%), H-4→L (6%), H-3→L (7%), H-1→L+1 (3%) |
| 6 | | 532.807 | | 2.327 | | 0.883 | | H-1→L+1 (77%), H→L+3 (11%), H-3→L+1 (3%), H-2→L (3%) |

**Table S8:** Wave length, excitation energy and oscillator strength of investigated compound **TPBR1** in gas phase at M06-2X/6-311 G(d,p) level.

| **No.** | **DFT λ (*nm*)** | **E(*eV*)** | ***f*_os_** | **MO contributions (%)** |
| --- | --- | --- | --- | --- |
| 1 | 547.488 | 2.265 | 1.920 | H→L (76%), H-2→L (5%), H-2→L+1 (9%), H-1→L (2%) |
| 2 | 479.333 | 2.587 | 0.351 | H-2→L (17%), H→L+1 (75%) |
| 3 | 426.737 | 2.905 | 0.133 | H-3→L (11%), H-3→L+1 (11%), H-1→L (61%), H→L (7%) |
| 4 | 408.098 | 3.038 | 0.188 | H-3→L (28%),H-1→L+1 (56%), H-3→L+1 (4%), H→L+1 (3%) |
| 5 | 363.686 | 3.409 | 0.017 | H-2→L (62%), H→L+1 (14%), H-4→L (3%), H-4→L+1 (4%), H-3→L (4%), H→L (5%) |
| 6 | 351.240 | 3.530 | 0.012 | H-4→L (14%),H-2→L+1 (33%), H-11→L (2%), H-11→L+1 (5%), H-11→L+3 (9%), H-3→L+1 (8%), H→L (3%), H→L+2 (6%), H→L+4 (3%) |

**Table S9:** Wave length, excitation energy and oscillator strength of investigated compound **TPBD2** in gas phase at M06-2X/6-311 G(d,p) level.

| **No.** | **DFT λ (*nm*)** | **E(*eV*)** | ***f*_os_** | **MO contributions (%)** |
| --- | --- | --- | --- | --- |
| 1 | 525.090 | 2.361 | 0.937 | H-1→L (19%), H→L (74%), H-3→L (3%) |
| 2 | 483.897 | 2.562 | 1.175 | H-3→L (11%), H-1→L (59%), H→L (24%) |
| 3 | 399.369 | 3.105 | 0.139 | H→L+1 (73%),H-3→L+1 (2%), H-1→L+1 (6%), H→L+2 (8%), H→L+3 (3%) |
| 4 | 397.245 | 3.121 | 0.379 | H-4→L (23%), H-3→L (14%), H-2→L (45%), H-5→L (3%), H-1→L+1 (2%), H→L+1 (3%) |
| 5 | 383.057 | 3.237 | 0.242 | H→L+2 (46%),H→L+3 (16%),H→L+4 (10%),H-3→L (4%), H-3→L+1 (2%),H-1→L+1(3%), H-1→L+2 (2%), H→L+1 (7%) |
| 6 | 376.829 | 3.290 | 0.066 | H-3→L+1 (20%), H-1→L+1 (46%), H→L+1 (16%),H-10→L+1 (3%), H-5→L+1 (3%) |

**Table S10:** Wave length, excitation energy and oscillator strength of investigated compound **TPBD3** in gas phase at M06-2X/6-311 G(d,p) level.

| **No.** | **DFT λ (*nm*)** | **E(*eV*)** | ***f*_os_** | **MO contributions (%)** |
| --- | --- | --- | --- | --- |
| 1 | 608.541 | 2.037 | 0.333 | H→L (92%), H-1→L (5%) |
| 2 | 534.668 | 2.319 | 1.472 | H-3→L (13%), H-1→L (70%), H-1→L+1 (2%), H→L (7%) |
| 3 | 446.532 | 2.777 | 0.106 | H→L+1 (86%), H-1→L+1 (6%), H→L+3 (3%) |
| 4 | 427.886 | 2.898 | 0.144 | H-4→L (19%), H-3→L (17%), H-2→L (35%), H-8→L (2%), H-5→L (6%), H-2→L+1 (4%), H-1→L+1 (7%) |
| 5 | 407.776 | 3.041 | 0.565 | H-3→L+1 (18%), H-1→L+1 (51%), H-5→L+1 (3%), H-4→L (3%), H-2→L (7%), H→L+1 (8%) |
| 6 | 388.799 | 3.189 | 0.224 | H-3→L(13%), H→L+3 (38%), H→L+4 (17%), H-3→L+1 (2%), H-1→L (3%), H-1→L+3 (2%), H→L+1 (3%), H→L+5 (2%), H→L+6 (8%) |

**Table S11:** Wave length, excitation energy and oscillator strength of investigated compound **TPBD4** in gas phase at M06-2X/6-311 G(d,p) level.

| **No.** | **DFT λ (*nm*)** | **E(*eV*)** | ***f*_os_** | **MO contributions (%)** |
| --- | --- | --- | --- | --- |
| 1 | 499.977 | 2.480 | 0.865 | H-1→L (19%), H→L (72%), H-3→L (3%), H→L+2 (2%) |
| 2 | 458.844 | 2.702 | 0.991 | H-3→L (13%), H-1→L (55%), H→L (26%) |
| 3 | 386.364 | 3.209 | 0.483 | H→L+1 (20%),H→L+2 (43%),H→L+3 (15%), H-1→L+1 (2%), H-1→L+2 (3%), H→L+4 (7%) |
| 4 | 383.413 | 3.234 | 0.054 | H-4→L (29%), H-3→L (10%), H-2→L (41%), H-8→L (3%), H-5→L (4%), H-1→L (4%) |
| 5 | 369.661 | 3.354 | 0.045 | H→L+1 (71%), H→L+2 (13%), H-1→L+1 (4%), H→L+3 (5%), H→L+4 (3%) |
| 6 | 350.317 | 3.539 | 0.167 | H-3→L+1 (15%), H-1→L+1 (48%), H-5→L+1 (2%), H-3→L (6%), H-2→L (3%), H-1→L+2 (6%), H→L+1 (7%) |

**Table S12:** Wave length, excitation energy and oscillator strength of investigated compound **TPBD5** in gas phase at M06-2X/6-311 G(d,p) level.

| **No.** | **DFT λ (*nm*)** | **E(*eV*)** | ***f*_os_** | **MO contributions (%)** |
| --- | --- | --- | --- | --- |
| 1 | 501.432 | 2.473 | 0.831 | H-1→L (18%), H→L (73%), H-3→L (3%) |
| 2 | 460.326 | 2.693 | 1.018 | H-3→L (14%), H-1→L (55%), H→L (25%), H-1→L+2 (2%) |
| 3 | 420.571 | 2.948 | 0.012 | H→L+1 (93%), H-1→L+1 (5%) |
| 4 | 388.397 | 3.192 | 0.160 | H-3→L+1 (20%), H-1→L+1 (51%), H-5→L+1 (3%), H→L+1 (7%), H→L+2 (5%), H→L+3 (2%) |
| 5 | 383.995 | 3.229 | 0.419 | H→L+2 (46%), H→L+3 (23%), H-3→L+1 (2%), H-2→L (2%), H-1→L+1 (5%), H-1→L+2 (2%), H→L+4 (9%) |
| 6 | 380.121 | 3.262 | 0.042 | H-4→L (26%), H-3→L (12%), H-2→L (38%), H-8→L (3%), H-5→L (4%), H-2→L+2 (2%), H-1→L (3%), H-1→L+1 (4%) |

**Table S13:** Wave length, excitation energy and oscillator strength of investigated compound **TPBD6** in gas phase at M06-2X/6-311 G(d,p) level.

| **No.** | **DFT λ (*nm*)** | **E(*eV*)** | ***f*_os_** | **MO contributions (%)** |
| --- | --- | --- | --- | --- |
| 1 | 596.078 | 2.080 | 0.341 | H→L (92%), H-1→L (6%) |
| 2 | 527.211 | 2.352 | 1.408 | H-3→L (13%), H-1→L (70%), H-1→L+1 (2%), H→L (7%) |
| 3 | 438.029 | 2.831 | 0.134 | H→L+1 (81%), H-1→L+1 (7%), H→L+3 (4%) |
| 4 | 423.472 | 2.928 | 0.153 | H-4→L (19%), H-3→L (16%), H-2→L (36%), H-8→L (2%), H-5→L (5%), H-2→L+1 (4%), H-1→L+1 (7%), H→L+1 (2%) |
| 5 | 402.481 | 3.081 | 0.543 | H-3→L+1 (17%), H-1→L+1 (50%), H→L+1 (11%), H-5→L+1 (3%), H-4→L (3%), H-2→L (7%) |
| 6 | 385.883 | 3.213 | 0.229 | H→L+3 (44%), H→L+4 (17%), H-3→L (9%), H-3→L+1 (2%), H-1→L+3 (3%), H→L+1 (4%), H→L+6 (9%) |

**Table S14:** Linear polarizabilities and major contributing tensors (*esu*) of the compounds **TPBR1** and **TPBD2**-**TPBD6**.

| **Compounds** | ***α_xx_* × 10^-22^** | ***α_yy_* × 10^-22^** | ***α_zz_* × 10^-22^** | **<α> × 10^-22^** |
| --- | --- | --- | --- | --- |
| **TPBR1** | 3.336 | 1.146 | 0.564 | 1.682 |
| **TPBD2** | 3.592 | 1.286 | 0.683 | 1.854 |
| **TPBD3** | 3.921 | 1.309 | 0.691 | 1.974 |
| **TPBD4** | 3.203 | 1.224 | 0.712 | 1.713 |
| **TPBD5** | 3.280 | 1.290 | 0.749 | 1.773 |
| **TPBD6** | 3.749 | 1.240 | 0.677 | 1.889 |

**Table S15:** Dipole moment (*u*) and major contributing tensors (*D*) of the studied compounds.

| **Compounds** | ***u*_xx_** | ***u*_yy_** | ***u*_zz_** | ***u*_total_** |
| --- | --- | --- | --- | --- |
| **TPBR1** | 7.640 | -4.816 | -1.801 | 9.208 |
| **TPBD2** | 13.085 | 0.614 | 0.496 | 13.108 |
| **TPBD3** | 16.447 | 2.539 | 1.222 | 16.686 |
| **TPBD4** | -9.221 | 1.446 | 3.160 | 9.854 |
| **TPBD5** | -9.795 | 0.793 | 3.432 | 10.408 |
| **TPBD6** | 16.295 | 2.213 | 1.026 | 16.476 |

**Table S16:** The computed first hyperpolarizabilities (*β*_tot_) and major contributing tensors (*esu*) of the studied compounds **TPBR1** and **TPBD2**-**TPBD6**.

| **Systems** | **TPBR1** | **TPBD2** | **TPBD3** | **TPBD4** | **TPBD5** | **TPBD6** |
| --- | --- | --- | --- | --- | --- | --- |
| ***β*_xxx_** | 0.020×10^-27^ | 1.969×10^-27^ | 3.894×10^-27^ | -1.576×10^-27^ | -1.678×10^-27^ | 3.467×10^-27^ |
| ***β*_xxy_** | 10.62×10^-29^ | -6.100×10^-29^ | 3.376×10^-29^ | 3.383×10^-29^ | 7.499×10^-29^ | -0.801×10^-29^ |
| ***β*_xyy_** | -0.183×10^-30^ | -2.123×10^-30^ | -5.408×10^-30^ | -2.465×10^-30^ | -2.988×10^-30^ | -2.354×10^-30^ |
| ***β*_yyy_** | 5.536×10^-30^ | 2.783×10^-30^ | 9.369×10^-30^ | -0.780×10^-30^ | -0.129×10^-30^ | 5.631×10^-30^ |
| ***β*_xxz_** | -4.241×10^-29^ | -0.173×10^-29^ | 3.177×10^-29^ | -2.234×10^-29^ | -2.379×10^-29^ | 1.442×10^-29^ |
| ***β*_yyz_** | -2.775×10^-30^ | 0.858×10^-30^ | 3.131×10^-30^ | 1.836×10^-30^ | 2.610×10^-30^ | 2.242×10^-30^ |
| ***β*_xzz_** | -3.714×10^-30^ | -4.058×10^-30^ | -4.030×10^-30^ | 0.914×10^-30^ | 1.293×10^-30^ | -3.866×10^-30^ |
| ***β*_yzz_** | 1.738×10^-30^ | 0.646×10^-30^ | 1.285×10^-30^ | -1.616×10^-30^ | -1.925×10^-30^ | 0.903×10^-30^ |
| ***β*_zzz_** | -0.227×10^-30^ | 2.074×10^-30^ | 2.336×10^-30^ | -2.135×10^-30^ | -1.889×10^-30^ | 2.275×10^-30^ |
| ***β*_total_** | **0.122×10^-27^** | **1.963×10^-27^** | **3.885×10^-27^** | **1.578×10^-27^** | **1.681×10^-27^** | **3.460×10^-27^** |

**Table S17:** Second hyperpolarizabilities and major contributing tensors (*esu*) of the studied compounds.

| **Compounds** | ***γ_x_*×10^-32^** | ***γ_y_*×10^-35^** | ***γ_z_*×10^-35^** | ***<γ>*×10^-32^** |
| --- | --- | --- | --- | --- |
| **TPBR1** | 1.028×10^-32^ | 8.608×10^-35^ | 19.04×10^-35^ | 1.039×10^-32^ |
| **TPBD2** | 3.425×10^-32^ | 13.02×10^-35^ | 0.593×10^-35^ | 3.439×10^-32^ |
| **TPBD3** | 12.99×10^-32^ | 25.27×10^-35^ | 1.099×10^-35^ | 13.02×10^-32^ |
| **TPBD4** | 2.412×10^-32^ | 9.306×10^-35^ | 2.787×10^-35^ | 2.424×10^-32^ |
| **TPBD5** | 2.666×10^-32^ | 8.889×10^-35^ | 2.641×10^-35^ | 2.677×10^-32^ |
| **TPBD6** | 10.83×10^-32^ | 1.590×10^-35^ | 0.723×10^-35^ | 10.85×10^-32^ |
